# Context-Dependent Anti-Predator Behavior in Nymphs of the Invasive Spotted Lanternfly (*Lycorma delicatula*): Effects of Development, Microhabitat, and Social Environment

**DOI:** 10.3390/insects16080815

**Published:** 2025-08-06

**Authors:** Ellen van Wilgenburg, Crystal Aung, Julia N. Caputo

**Affiliations:** Department of Natural Sciences, Fordham University, New York, NY 10023, USA

**Keywords:** aposematism, antipredator behavior, escape, spotted lanternfly, ontogenetic color change, instar

## Abstract

Antipredator behaviors in animals often vary with developmental stage, microhabitat, and social context, yet few studies examine how these factors interact in species that undergo ontogenetic shifts in chemical defense. The spotted lanternfly (*Lycorma delicatula*) is an invasive planthopper whose nymphs transition from cryptically colored early instars to aposematically colored fourth instars that feed primarily on chemically defended host plants. We conducted 1460 simulated predator attacks on nymphs across four developmental stages to examine how antipredator behavior varies with instar, plant location (leaf vs. stem), host plant species, and local conspecific density. Nymphs exhibited three primary responses: hiding, sidestepping, or jumping. We found that location on the plant had the strongest effect, with nymphs on stems more likely to hide than those on leaves. Older instars were significantly less likely to hide and more likely to sidestep, particularly on stems, suggesting reduced reliance on energetically costly escape behaviors as chemical defenses accumulate. First instars were less likely to jump from their preferred host plant (tree of heaven) compared to other plant species. Higher local conspecific density reduced hiding probability, likely due to the dilution effect. These results demonstrate that antipredator strategies in *L. delicatula* are flexibly deployed based on developmental stage, microhabitat structure, and social context, with implications for understanding evolution of antipredator behavior in chemically protected species.

## Simple Summary

The spotted lanternfly is an invasive insect from China that is rapidly increasing its distribution in the United States. Spotted lanternfly nymphs go through four developmental stages, changing color from black with white spots to bright red as they mature. Understanding how these insects avoid predators could help scientists develop better control strategies. This study examined how spotted lanternfly nymphs react when threatened by predators. We simulated predator attacks and recorded three main responses: hiding behind plant parts, sidestepping, or jumping. The results showed that nymphs on stems were much more likely to hide than those on leaves, but they became less likely to hide and more likely to sidestep as they became older. Young nymphs were more likely to jump from plants that were not tree of heaven, their preferred host plant. When many spotted lanternflies nymphs were clustered together, individuals were less likely to hide, possibly because of perceived safety in numbers. These findings reveal that spotted lanternflies use flexible defense strategies that change as they develop. This knowledge could help pest control experts predict where and when these insects are most vulnerable, leading to more targeted management approaches that protect agricultural crops and native ecosystems.

## 1. Introduction

Animals have evolved a wide range of behaviors and traits to reduce the risk of predation, either by minimizing the chance of being detected (e.g., through camouflage or crypsis) or, if detected, by avoiding capture (e.g., by fleeing of fighting back) [1,2,3]. A single individual often possesses multiple types of antipredator responses, and the response it exhibits in a given situation may depend on internal factors such as age [4,5,6], sex [5,7], and nutritional condition [8], as well as external factors such as habitat structure [9], the presence of conspecifics [10], and the nature of the predator [11].

Two common antipredator strategies, cryptic coloration and aposematic coloration, represent contrasting evolutionary solutions to the threat of predation. Cryptic species use coloration and patterning to blend into their environments, reducing the likelihood of detection by predators. When detected, these species typically rely on rapid escape behaviors to avoid capture [12,13], thus conserving energy by avoiding unnecessary movement unless a threat is imminent. In contrast, aposematic species advertise their unpalatability or toxicity through conspicuous coloration [14,15]. This warning signal deters predators from attacking, leading to lower rates of predation. As a result, aposematic prey often exhibit slower, more predictable responses when confronted by predators [16,17,18], and they can afford to forage more openly, even in the presence of predators [19].

**Figure 1:**
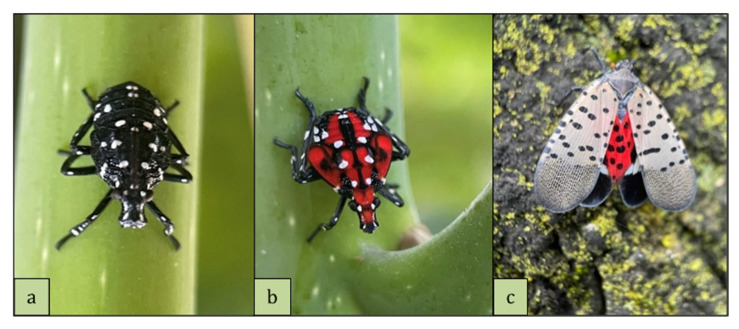
(**a**) 3rd instar, (**b**) 4th instar and (**c**) adult spotted lanternfly.

The spotted lanternfly (SLF) (*Lycorma delicatula*) is a planthopper native to China, Japan and Vietnam that has recently established populations in the United States [20,21,22]. It exhibits a univoltine life cycle: after hatching from eggs in the spring, individuals pass through four distinct nymphal instars, each lasting approximately two weeks [21,23,24,25]. The first three instars are black with white spots, while the fourth instar is conspicuously red with white and black markings (Figure 1a,b). Adult lanternflies emerge in the summer and have cryptically colored forewings that conceal bright red hindwings (Figure 1c). In the USA, *L. delicatula* has relatively few natural predators, but a growing number of generalist predators, including arthropods and birds, are now feeding on *L. delicatula* [26].

Adult *L. delicatula* exhibit a variety of antipredator behaviors, including jumping, deimatic (startle) displays, and death-feigning [27,28,29]. The expression of antipredator behaviors varies with both the sex of the individual and the timing within the mating season [28,29]. Females consistently perform deimatic displays and are more likely to jump away in response to repeated attacks, whereas some males do not exhibit deimatic displays at all. As the mating season progresses, individuals transition from predominantly employing a deimatic-escape strategy in early adulthood to increasingly relying on deimatic-warning and death-feigning behaviors in later adulthood, with a marked increase in death-feigning frequency over time. Although less is known about predator responses in the nymphal stages, nymphs demonstrate powerful jumping abilities, which likely serve as an effective escape mechanism [30,31].

During the first three instars, nymphs feed on the phloem of a wide range of host plants spanning 18 families [20]. However, in the fourth instar, marked by a red aposematic coloration, they switch almost exclusively to feeding on *Ailanthus altissima* (tree of heaven, TOH), a plant rich in quassinoids and alkaloids [32,33]. A prior study [34] found that feeding on *A. altissima* renders SLFs increasingly unpalatable with age, and birds exhibit learned taste aversion to individuals collected from this host. This ontogenetic shift in host preference, coinciding with the acquisition of warning coloration, likely represents an honest signal of chemical defense [35].

While studies have highlighted the differences in antipredator behavior between generally aposematic and cryptic species, research examining how antipredator behavior may shift in response to intraspecific ontogenetic (developmental) changes in unpalatability and coloration is comparatively scarce. This area is crucial for understanding the adaptive strategies animals employ across their life stages and how a suite of defensive traits can evolve and be deployed sequentially or simultaneously during the predation sequence. In the present study, we conducted a field experiment in which we simulated predatory attacks to determine how antipredator behavior varies among nymphal stages, species of host plant and location of the SLF on the host plant. Preliminary observations suggest that nymphs employ three main tactics when disturbed; sidestepping along the substrate, hiding behind plant structures, and jumping off the plant. However, how the choice among these responses varies with ontogeny, location on the plant and social context is unknown.

This study had three specific aims. First, we aimed to characterize how developmental stage (1st–4th instar) influences the relative use of sidestepping, hiding, and jumping. We hypothesized that younger instars would predominantly rely on jumping, whereas older, presumably chemically defended, instars would more frequently employ sidestepping behaviors. Second, we tested whether microhabitat (leaf vs. stem surface) and host-plant identity (tree-of-heaven, the primary host, vs. other plant species) modulate these behaviors, both independently and in interaction with instar stage. We predicted that individuals on stems would be more likely to hide, while those on leaves would jump more often. We also predicted that nymphs on plants other than their preferred host plant (*A. altissima*) would jump more frequently. Finally, we aimed to quantify the effect of local conspecific density on antipredator decisions, hypothesizing that individuals in higher-density areas would exhibit reduced escape responses due to perceived safety in numbers.

## 2. Materials and Methods

We examined antipredator behavior in *L. delicatula* nymphs through simulated predator attack trials conducted on wild individuals in Central Park, New York City. Behavioral assays were performed weekly from 19 May to 24 July 2023, across multiple host plants commonly used by *L. delicatula*, including *Ailanthus altissima* (tree-of-heaven), crabapple (*Malus* spp.), cork trees (*Phellodendron* spp.), and American beech (*Fagus grandifolia*).

To simulate a predator strike, we approached each nymph using the blunt end of a fine paintbrush and delivered a gentle tap from above the insect perpendicular to the substrate surface in the direction of the body. This method was designed to mimic a pecking motion, based on prior work suggesting birds are primary predators of *L. delicatula* [26,28,29]. For each trial, we recorded one of three behavioral responses: (1) hiding—the nymph swiftly moved to the opposite side of the leaf or stem, out of reach of the brush and out of the experimenter’s view; (2) side-stepping—a small (<1 cm) lateral movement that avoided contact with the paintbrush without fully fleeing out of sight; and (3) jumping—the nymph launched itself from the substrate, often leaving the plant entirely. Before each assay, we counted the number of surrounding spotted lanternflies within a 10 cm radius of the focal nymph.

We conducted a total of 1460 trials. We balanced assays between plant parts (fleshy stems vs. leaves) and host types, performing roughly equal numbers on A. altissima and on other host species (pooled). To prevent pseudoreplication and minimize disturbance effects, only one individual was tested per plant, and trials were spaced to avoid influencing nearby individuals.

### Statistical Analysis

We used multinomial logistic regression models to model the probability of each antipredator behavior as a function of the predictor variables and all interactions. Sidestep was used as the reference category. The model was fit using maximum likelihood estimation with the Newton-Raphson algorithm [36,37]. Model selection was performed using stepwise addition of two-way interaction terms, with Akaike Information Criterion (AIC) used to identify the best-fitting model [38,39]. The full model included main effects of instar stage (1st–4th), host-tree category (tree of heaven vs. other species), plant location (leaf vs. stem), and local conspecific density, along with two-way interactions between instar and plant location, and instar and tree category. The model converged successfully under maximum likelihood estimation using the Newton-Raphson algorithm (6 iterations, n = 1460) and demonstrated significant overall fit (LLR χ^2^ = 482.57, df = 24, *p* < 0.001, Pseudo R^2^ = 0.154). Effect sizes were interpreted using the magnitude of odds ratios (OR). An OR of 1 means no change in the odds, less than 1 indicates a decrease, and greater than 1 indicates an increase. For instance, an OR of 1.25 suggests a 25% increase in the odds a nymph hides [40]. Interaction effects were interpreted by examining the modification of main effects across levels of the interacting variables, with significant interactions indicating that the effect of one predictor depends on the level of another predictor.

We calculated predicted probabilities for each behavioral outcome from the final model and visualized them across key predictor combinations to illustrate the effects of developmental stage, environment, and group size on antipredator behavior. All analyses were conducted using Python 3.11.6 with the statsmodels package (version 0.14.0) for multinomial logistic regression fitting and diagnostic procedures. Data analysis was conducted with the help of Julius AI, an AI assistant powered by GPT-4 and designed for data science applications. The assistant was utilized within a Jupyter notebook environment to perform statistical analysis and visualization generation. To verify the accuracy and reproducibility of the AI-generated results, we reviewed the code, made all data publicly available, checked whether the conclusions logically followed from the data, compared the AI’s output with results obtained using SPSS 29, repeated the analysis multiple times, and looked for any logical errors.

## 3. Results

We observed a total of 1460 Spotted Lanternfly nymphs across four instar stages. The overall distribution showed hiding as the most frequent response (43.7%), followed by sidestepping (31.7%) and jumping (24.6%).

### 3.1. Multinomial Regression Results

**Figure 2:**
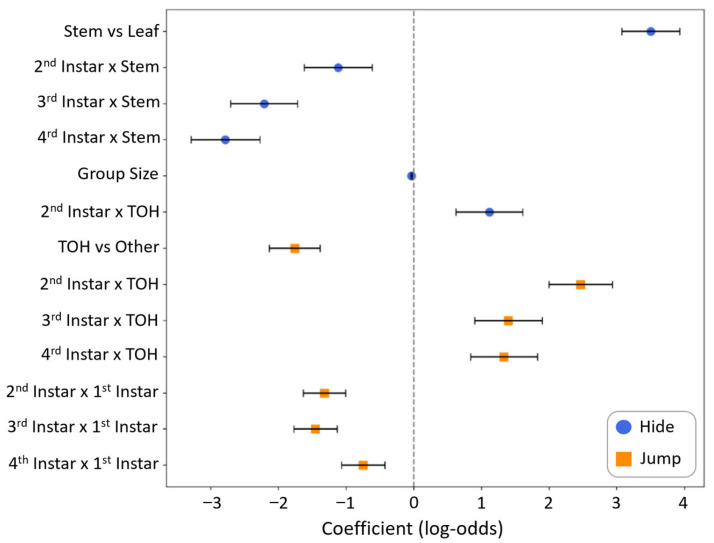
Model coefficients and standard errors for predictors of antipredator behavior in SLF nymphs. Each point represents a model coefficient (log-odds), with horizontal error bars indicating the standard error. Predictors with positive coefficients increase the odds of the focal behavior, while negative coefficients decrease it.

The model identified several key predictors and interactions that significantly influenced the likelihood of hiding and jumping behaviors compared to sidestepping (Figure 2) as shown below. The full model can be found in the Supplementary Materials.

#### 3.1.1. Location on Plant and Developmental Effects

Location of the nymph on a plant had the strongest effect on the type of behavior the nymphs displayed in response to the simulated predator attack. The likelihood of hiding was significantly higher on stems compared to leaves (β = 3.50, SE = 0.43, z = 8.17, *p* < 0.001, OR = 33.12). However, this effect was significantly reduced for older instars, as indicated by negative interactions for 2nd instar × stem (β = −1.12, SE = 0.50, z = −2.22, *p* = 0.026, OR = 0.33), 3rd instar × stem (β = −2.21, SE = 0.49, z = −4.48, *p* < 0.001, OR = 0.11), and 4th instar × stem (β = −2.78, SE = 0.51, z = −5.48, *p* < 0.001, OR = 0.06). These interactions indicate that while 1st instar nymphs strongly favor hiding when on stems, this preference diminishes markedly with development. In contrast, stem location had no significant effect on jumping behavior (*p* > 0.05).

Instar stage demonstrated differential effects on behavior choice. Relative to 1st instars, all later developmental stages showed significantly reduced jumping behavior: 2nd instars (β = −1.32, SE = 0.31, z = −4.23, *p* < 0.001, OR = 0.27), 3rd instars (β = −1.46, SE = 0.32, z = −4.55, *p* < 0.001, OR = 0.23), and 4th instars (β = −0.75, SE = 0.32, z = −2.33, *p* = 0.020, OR = 0.47). Main effects of instar on hiding behavior were not statistically significant (*p* > 0.05)

#### 3.1.2. Host Plant Effects

Nymphs were significantly less likely to jump from a tree of heaven compared to other plant species (β = −1.76, SE = 0.37, z = −4.70, *p* < 0.001, OR = 0.17). However, this effect was offset for older instars by positive interaction terms: 2nd instar × TOH (β = 2.46, SE = 0.47, z = 5.24, *p* < 0.001, OR = 11.74), 3rd instar × TOH (β = 1.40, SE = 0.50, z = 2.81, *p* = 0.005, OR = 4.04), and 4th instar × TOH (β = 1.33, SE = 0.49, z = 2.69, *p* = 0.007, OR = 3.79). These interactions suggest that while tree of heaven generally suppresses jumping behavior, this suppression weakens in older instars, with 2nd instars showing the most pronounced recovery.

#### 3.1.3. Group Size Effects

Local conspecific density, estimated as the number of other nymphs within 10 cm, showed a significant negative association with hiding behavior (β = −0.034, SE = 0.010, z = −3.28, *p* = 0.001, OR = 0.97), suggesting that the odds of hiding decreased by 3% for every additional nearby nymph within 10 cm. The effect of local conspecific density on jumping behavior was not statistically significant (*p* > 0.05).

### 3.2. Predicted Probabilities Across Conditions

**Figure 3:**
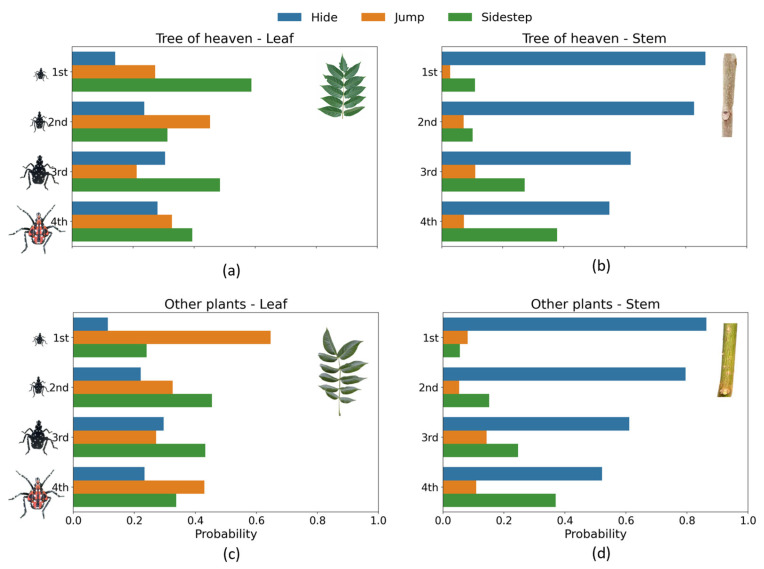
Predicted probabilities of each antipredator behavior on (**a**) leaves of tree of heaven, (**b**) stems of tree of heaven, (**c**) leaves of other plants, and (**d**) stems of other plants.

To illustrate the effects of different factors on antipredation behavior and to make the complex interactions between factors more visible, we calculated predicted probabilities from the final model and plotted them across instars, tree categories, and microhabitats (Figure 3). On stems of TOH and other plant species, hiding dominated across all instar stages, especially for younger instars. The predicted probability of hiding on stems of TOH decreases from 73.1 to 36.5% while the predicted probability of sidestepping increases from 23.6 to 57.0% between 1st and 4th instar. A similar pattern was found for nymphs on stems of other plants.

1st instars are less likely to jump from leaves of TOH compared to leaves from other plants (20.4% vs. 56.8) On stems, the differences are smaller, but TOH still tends to reduce jumping behavior in 1st instars (3.2 vs. 10.2%).

**Figure 4:**
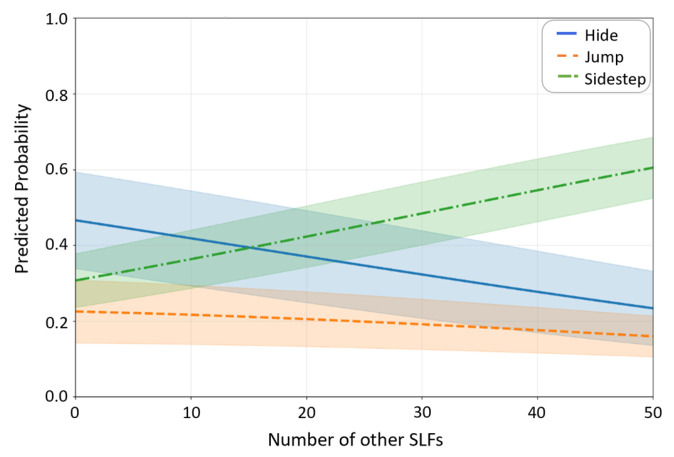
Overall effect of the number of other SLF nymphs within a 10 cm radius on predicted probabilities for each antipredator behavior, averaged across all developmental stages, tree categories, and microhabitats. The shaded ribbons represent 95% confidence intervals around each prediction.

When averaged across all developmental stages, tree categories, and microhabitats, the group size effect was pronounced (Figure 4). As the number of other SLF nymphs increased from 0 to 50, the probability of sidestepping rose from 31% to 61%, while hiding dropped from 47% to 23%, and jumping decreased from 23% to 16%.

## 4. Discussion

Our study reveals that antipredator behaviors of SLF nymphs depend on developmental stage, location on a host plant, the number of other nymphs in the vicinity, and species of host plant, with these factors often interacting. These findings highlight the complex and context-dependent nature of antipredator strategies throughout an insect’s development. These findings are particularly notable in this system given that SLF nymphs shift from cryptic to aposematic coloration, with accompanying changes in chemical defenses, through ontogeny.

One of the key findings of our study is that microhabitat structure, specifically location on the host plant, strongly shapes antipredator behavior in SLF nymphs. Nymphs on stems were more likely to hide than those on leaves in response to a simulated predator. This pattern likely reflects the relative costs and benefits of each behavior: hiding behind a stem requires minimal movement and offers quick concealment, while jumping from a leaf—where hiding may demand more time or exposure—provides a faster escape. These findings align with broader evidence that structural features of an organism’s environment, like distance to shelter, influence escape behaviors in animals as diverse as fish, lizards and rodents [41]. For instance, Schulte et al. [42] found that the presence and characteristics of refuges play a crucial role in escape decisions in lizards.

The higher frequency of nymph jumping from leaves compared to stems may also reflect differences in outcomes and associated costs of the jumping strategy. These costs and benefits have been examined in a prior study of antipredator behavior in beetles, which showed that when these insects inhabit plants with overlapping leaves, they typically drop onto lower leaves rather than dropping on the ground, which reduces both risk exposure and energy costs [9]. Similarly, SLF nymphs launching from leaves may have a greater probability of landing on foliage instead of the ground while nymphs jumping from vertical stems face an increased likelihood of ending up on the ground. Though SLF nymphs demonstrate rapid, long-distance jumping behavior [31], we did not measure whether leaf-based jumpers actually achieve more successful foliage landings compared to stem-based jumpers, but this could be the subject of a future study.

Alternatively, nymphs may be less prone to jump from stems because stems are higher-quality feeding sites for phloem feeders [43]. Staying put can be favored when the cost of abandoning a high-quality resource (e.g., nutritious food, rare host plants) outweighs the potential benefit of escaping. Juvenile crayfish, for example, exhibit similar trade-offs, choosing to freeze near food rather than escape and lose access to a valuable resource [44]. Across systems, these examples underscore that antipredator behavior is not fixed but instead dynamically shaped by microhabitat context, with organisms selecting strategies that best fit the immediate constraints of their environment.

Beyond location, nymphal stage also played a crucial role in shaping behavioral responses, particularly for nymphs on stems. While the behavior of nymphs on leaves remained relatively consistent across instars, later instars on stems became significantly less likely to hide and more likely to sidestep compared to earlier instars. Sidestepping involves only a small movement, and we hypothesize that this behavior represents a less energetically costly behavior than hiding or jumping.

These ontogenetic shifts in behavior are likely linked to the accumulation of chemical defenses from their host plants. Whilst our study did not directly measure chemical defense enhancement, research indicates that the tree of heaven, a primary host for SLF, provides defensive chemicals, specifically quassinoids like ailanthone, that render the lanternflies unpalatable to birds [34]. Importantly, a previous study has shown that the unpalatability of SLF increases with age, especially when feeding on tree of heaven [34]. Chemical analyses show that nymphs feeding on tree of heaven gradually build up concentrations of these defensive compounds in their bodies [34]. The observed shift towards potentially less energetically costly antipredator responses like sidestepping in older nymphs on stems may reflect a decreased reliance on active evasion as their increasing unpalatability offers a passive defense. It is noteworthy that the gradual change in behavior observed throughout SLF development (Instars 1–4) is not solely tied to the distinct color change from the black and white early instars to the aposematic red fourth instar. While conspicuous coloration is a key component of aposematism, signaling unpalatability, the acquisition of unpalatability appears to be a more continuous process linked to feeding on the host plant over time. The findings here suggest that behavioral adjustments to predator threat are influenced by this continuous process rather than solely by the visual signal of the final nymphal stage. Ontogenetic shifts in unpalatability have been shown to be accompanied by changes in behavior in other insects. For example, the panic moth (*Saucrobotys futilalis*) transitions from cryptic green early instars to aposematic, unpalatable later instars. As chemical defenses and visual warning signals develop, caterpillars move from hiding within leaf shelters to more conspicuous foraging and defensive behaviors like regurgitation and immobility, reflecting a shift from reliance on crypsis to active chemical and behavioral defense [6].

Interestingly, SLF nymphs located on host plants other than tree of heaven exhibited similar ontogenetic behavioral shifts, also becoming significantly less likely to hide and more likely to sidestep in later instar stages. This pattern suggests that the behavioral changes observed may not be exclusively dependent on the accumulation of quassinoids from tree of heaven. Several explanations could account for this phenomenon. First, SLF nymphs are known to be highly mobile and may move between different host plant species during their development. Nymphs feeding on alternative hosts such as grapevine, black walnut, or other preferred species may periodically return to tree of heaven or encounter it during dispersal, potentially allowing for some accumulation of defensive compounds even when primarily associated with other host plants. Second, alternative host plants may themselves contain defensive compounds that SLF nymphs can sequester and accumulate over time. Many plant species produce secondary metabolites such as alkaloids, phenolics, or other bioactive compounds [45,46] that could provide similar antipredator benefits to the quassinoids found in tree of heaven. The uptake of these alternative defensive chemicals could similarly lead to increased unpalatability and the corresponding shift toward less energetically costly antipredator behaviors. Alternatively, the ontogenetic shift toward potentially less energetically costly antipredator behaviors like sidestepping may represent an intrinsic developmental pattern that is not directly correlated with the uptake of any defensive compounds. This behavioral transition could be driven by other age-related factors such as changes in body size and mobility, which occur independently of chemical defense acquisition. Controlled experiments comparing nymphs reared exclusively on tree of heaven versus those maintained solely on alternative host plants throughout their development would help distinguish between these competing hypotheses.

The influence of the host plant was particularly pronounced in the youngest nymphs. First instar nymphs were significantly less likely to jump off tree of heaven compared to other plants. This behavior is not seen in later instars. This may be adaptive for first instars, which are small and benefit greatly from acquiring the defensive chemicals from tree of heaven early in their development. Jumping from the host plant, especially one that provides essential defensive compounds, carries the risk of failing to return and locate a suitable food source. As nymphs grow and potentially become more mobile or accumulate sufficient defenses, this constraint may lessen, explaining the lack of host plant effect on jumping in later instars.

Congregating with conspecifics provides several antipredation advantages [41], but crowding can also constrain particular antipredator behaviors [47]. Our model revealed that local crowding, measured as the number of other spotted-lanternfly nymphs within 10 cm, selectively alters escape tactics. We found that the odds of hiding decreased by 3% for every additional nearby nymph within 10 cm. Hiding, by circling around the stem or moving to the opposite side of a leaf, involves a nymph simply rotating to keep the stem or leaf between itself and an approaching threat. Unlike hiding in crevices, this behavior is limited less by refuge availability than by spatial interference from neighbors. However, when stems or leaves become crowded, moving to the “blind” side may be harder, potentially explaining the negative group-size coefficient. As nymphs develop through successive molts, they undergo substantial growth, with fourth instar nymphs exceeding three times the body length of first instars [48]. Given that larger nymphs occupy considerably more space than younger stages, they should theoretically experience spatial constraints even at relatively low population densities. If crowding were the primary driver of behavioral changes, we would have expected to see a significant effect of the interaction between developmental stage and local density on antipredator behavior. The absence of such an effect suggests that factors beyond physical crowding may be responsible for the observed behavioral shifts across instars. Being in a group offers clear antipredator benefits. The dilution effect lowers the individual probability of being attacked as predator when attention is divided among many potential targets [49,50]. This benefit likely offset the cost of abandoning the hiding maneuver, leading to the observed shift towards sidestepping at higher densities. The independence of jumping from group size suggests that this energetically expensive, high-risk tactic is triggered by immediate threat proximity rather than by social cues. It should be noted that our analysis was observational rather than experimental, and a future study in which nymph density is experimentally controlled may shed more light on these density-behavior relationships.

Prior work has suggested that birds are primary predators of *L. delicatula* [26,28,29], and we found that hiding was the most frequent response of nymphs to simulated bird attacks, followed by sidestepping and jumping. Different predators likely trigger distinct behavioral responses in their prey, meaning our assay may have provoked a different set of defensive behaviors than would occur during attacks by, for example, spiders or praying mantids. Additionally, the developmental changes in antipredator behavior observed across instars may vary depending on the predator type, as different predators may show varying sensitivity to the toxic compounds that nymphs produce.

Our findings reveal the complexity and context-dependency of antipredator behaviors in a single insect species. The variation across nymphal stages, influenced by both their changing physiology (acquisition of defenses) and their color, combined with the fine-scale environmental cues of location on the plant and the broad-scale effect of host plant species (for young nymphs), paints a picture of a flexible and adaptive defense repertoire. Future research should aim to quantify the energetic costs associated with different antipredator behaviors (jumping, hiding, sidestepping) to formally test the hypothesis that shifts occur towards less costly behavioral strategies as chemical defense increases.

## Data Availability

Data supporting reported results can be found in Dryad. https://doi.org/10.5061/dryad.qrfj6q5v4 (accessed on 20 July 2025).

## Supplementary Materials

The following are available online at https://www.mdpi.com/article/10.3390/insects16080815/s1, Table S1: Multinomial logistic regression coefficients for spotted lanternfly behavioral responses.
