# Supplementary material for: Context-Dependent Anti-Predator Behavior in Nymphs of the Invasive Spotted Lanternfly (Lycorma delicatula): Effects of Development, Microhabitat, and Social Environment
Source: Insects. 2025 Aug 6;16(8):815. doi: 10.3390/insects16080815 (PMC12386867; doi:10.3390/insects16080815)
Supplement: Supplementary file 1 [file insects-16-00815-s001.zip › insects-3792352-supplementary.pdf]

Supplementary Table S1. Multinomial logistic regression coefficients for spotted lanternfly behavioral responses

Comparison	Predictor Variable	Coefficient	Standard Error	z-value	p-value	95% Confidence Interval
Hide vs Sidestep	Intercept	-0.755	0.292	-2.58	0.010	[-1.328, -0.182]
Hide vs Sidestep	Instar (2nd)	0.033	0.385	0.08	0.932	[-0.722, 0.787]
Hide vs Sidestep	Instar (3rd)	0.377	0.371	1.02	0.309	[-0.35, 1.104]
Hide vs Sidestep	Instar (4th)	0.386	0.394	0.98	0.327	[-0.385, 1.158]
Hide vs Sidestep	Treecat (TOH)	-0.672	0.401	-1.68	0.094	[-1.458, 0.114]
Hide vs Sidestep	Leaf or Stem (Stem)	3.5	0.429	8.17	3.18e-16	[2.66, 4.34]
Hide vs Sidestep	Instar (2nd) Ã— Leaf or Stem (Stem)	-1.118	0.503	-2.22	0.026	[-2.104, -0.132]
Hide vs Sidestep	Instar (3rd) Ã— Leaf or Stem (Stem)	-2.213	0.494	-4.48	7.41e-06	[-3.181, -1.245]
Hide vs Sidestep	Instar (4th) Ã— Leaf or Stem (Stem)	-2.785	0.509	-5.48	4.35e-08	[-3.781, -1.788]
Hide vs Sidestep	Instar (2nd) Ã— Treecat (TOH)	1.114	0.495	2.25	0.024	[0.144, 2.084]
Hide vs Sidestep	Instar (3rd) Ã— Treecat (TOH)	0.587	0.476	1.23	0.217	[-0.345, 1.52]

Comparison	Predictor Variable	Coefficient	Standard Error	z-value	p-value	95% Confidence Interval
Hide vs Sidestep	Instar (4th) Ã— Treecat (TOH)	0.699	0.469	1.49	0.136	[-0.22, 1.618]
Hide vs Sidestep	Number other SLF	-0.034	0.01	-3.28	0.001	[-0.055, -0.014]
Jump vs Sidestep	Intercept	0.99	0.204	4.85	1.23e-06	[0.59, 1.389]
Jump vs Sidestep	Instar (2nd)	-1.324	0.313	-4.23	2.30e-05	[-1.937, -0.711]
Jump vs Sidestep	Instar (3rd)	-1.455	0.32	-4.55	5.44e-06	[-2.082, -0.828]
Jump vs Sidestep	Instar (4th)	-0.749	0.321	-2.33	0.020	[-1.377, -0.12]
Jump vs Sidestep	Treecat (TOH)	-1.76	0.375	-4.7	2.62e-06	[-2.494, -1.026]
Jump vs Sidestep	Leaf or Stem (Stem)	-0.616	0.546	-1.13	0.259	[-1.686, 0.453]
Jump vs Sidestep	Instar (2nd) Ã— Leaf or Stem (Stem)	-0.092	0.65	-0.14	0.888	[-1.366, 1.182]
Jump vs Sidestep	Instar (3rd) Ã— Leaf or Stem (Stem)	0.54	0.622	0.87	0.385	[-0.679, 1.759]
Jump vs Sidestep	Instar (4th) Ã— Leaf or Stem (Stem)	-0.845	0.63	-1.34	0.180	[-2.081, 0.39]
Jump vs Sidestep	Instar (2nd) Ã— Treecat (TOH)	2.463	0.47	5.24	1.58e-07	[1.542, 3.384]

Comparison	Predictor Variable	Coefficient	Standard Error	z-value	p-value	95% Confidence Interval
Jump vs Sidestep	Instar (3rd) Ã— Treecat (TOH)	1.396	0.498	2.81	0.005	[0.421, 2.372]
Jump vs Sidestep	Instar (4th) Ã— Treecat (TOH)	1.332	0.494	2.69	0.007	[0.363, 2.301]
Jump vs Sidestep	Number other SLF	-0.02	0.016	-1.25	0.210	[-0.052, 0.011]

Table Legend: Results from multinomial logistic regression analysis examining factors influencing spotted lanternfly (SLF) behavioral responses. The reference category for behavioral comparisons is "Sidestep." Coefficients represent the log-odds of the specified behavior relative to sidestepping. Predictor variables include developmental instar stage (1st instar as reference), tree category (Tree of Heaven vs. other trees), substrate type (leaf vs. stem), and number of other SLF nymphs present within a 10cm radius. Interaction terms (Ã—) indicate multiplicative effects between predictor variables. Standard errors (SE), z-values, p-values, and 95% confidence intervals are provided for each coefficient. Significant effects ($p < 0.05$) indicate predictors that significantly influence the likelihood of the specified behavioral response compared to sidestepping.
